# Adiponectin protects against paraquat-induced lung injury by attenuating oxidative/nitrative stress

**DOI:** 10.3892/etm.2014.2073

**Published:** 2014-11-17

**Authors:** RONG YAO, YAXIONG ZHOU, YARONG HE, YAOWEN JIANG, PENG LIU, LEI YE, ZHIJIE ZHENG, WAYNE BOND LAU, YU CAO, ZHI ZENG

**Affiliations:** 1 Department of Emergency Medicine, West China Hospital, Sichuan University, Chengdu, Sichuan 610041, P.R. China; 2 Electrocardiogram Department, No.4 West China Teaching Hospital, Sichuan University, Chengdu, Sichuan 610041, P.R. China; 3 Department of Emergency Medicine, Thomas Jefferson University Hospital, Philadelphia, PA 19107, USA

**Keywords:** globular adiponectin, paraquat, lung injury, oxidative stress, nitrative stress

## Abstract

The specific mechanisms underlying paraquat (PQ)-induced lung injury remain unknown, which limits understanding of its cytotoxic potential. Although oxidative stress has been established as an important mechanism underlying PQ toxicity, multiple antioxidants have proven ineffective in attenuating the deleterious effects of PQ. Adiponectin, which shows anti-oxidative and antinitrative effects, may have the potential to reduce PQ-mediated injury. The present study determined the protective action of globular domain adiponectin (gAd) on PQ-induced lung injury, and attempted to elucidate the underlying mechanism or mechanisms of action. BALB/c mice were administered PQ, with and without 12 or 36 h of gAd pre-treatment. The pulmonary oxidative/nitrative status was assessed by measuring pulmonary O_2_^•−^, superoxide dismutase (SOD), malondialdehyde (MDA), nitric oxide (NO) and 8-hydroxy-2-dydeoxy guanosine (8-OHdG) production, and blood 3-Nitrotyrosine (3-NT). At a dose of 20 mg/kg, PQ markedly increased O_2_^•−^, SOD, MDA, NO and 8-OHdG production 3 h post-administration, but did not significantly increase 3-NT levels until 12 h. gAd inhibited these changes in a dose-dependent manner, via transient activation of MDA, followed by attenuation of MDA formation from 6 h onwards. Histological analysis demonstrated that gAd decreased interstitial edema and inflammatory cell infiltration. These results suggest that gAd protects against PQ-induced lung injury by mitigating oxidative/nitrative stress. Furthermore, gAd may be a potential therapeutic agent for PQ-induced lung injury, and further pharmacological studies are therefore warranted.

## Introduction

Paraquat (1,1′-dimethyl-4,4′-bipyridinium dichloride; PQ) is a widely-utilized non-selective herbicide for the control of broadleaf weed. Available therapies for PQ poisoning lack efficacy, and it is a major cause of herbicide-related mortality, which occurs due to respiratory failure secondary to lung injury (1). Although the specific mechanisms of PQ toxicity have not been fully defined, it is hypothesized that PQ toxicity involves the generation of reactive oxygen species (ROS), leading to subsequent oxidative stress, which results in cell death and lung tissue damage (1,2). Antioxidants that directly inhibit ROS generation have been proposed as possible therapeutic agents in PQ-induced lung injury. However, traditional antioxidants, such as glutathion and vitamin C, have failed to protect against the characteristic pathological lung changes or mortality observed in PQ poisoning (1). Therefore, mechanisms other than oxidative stress may also be responsible for the pulmonary toxicity of PQ. The identification of such mechanisms may yield insight into the development of novel therapeutic agents and is an important area of research.

Adiponectin (also termed Acrp30, AdipoQ and GBP28) is a protein predominantly secreted from adipose tissue (3–5). Primary sequence analysis has revealed that full-length adiponectin has four main domains, with the globular segment (gAd) at the carboxy terminus being much more potent than the full protein (6,7). Adiponectin has been demonstrated to exhibit anti-oxidative and anti-inflammatory effects (8). The mechanisms of its actions are varied and depend upon the site of activity. In endothelial cells, adiponectin enhances nitric oxide (NO) production, suppresses the production of ROS and protects against inflammation mediated by hyperglycemic states or tumor necrosis factor, via activation of AMP-activated protein kinase and cyclic AMP-dependent protein kinase signaling cascades (9). It was hypothesized that globular domain adiponectin (gAd) may protect against PQ-induced lung injury by attenuating oxidative/nitrative stress.

The present study aimed to determine whether gAd protects against PQ-induced pulmonary injury in BALB/c mice and to determine the underlying mechanism or mechanisms of action. Ultimately the aim would be to assess the potential of adiponectin as a novel therapeutic agent in PQ-induced lung injury.

## Materials and methods

### Materials

PQ was obtained from Tokyo Kasei Kogyo Co., Ltd. (Tokyo, Japan). Recombinant mouse gAd was obtained from Adipobiotech Inc. (Beijing, China). Enzyme-linked immunosorbent assay (ELISA) kits for 3-Nitrotyrosine (3-NT) were obtained from Abcam (Cambridge, MA, USA). 8-hydroxy-2-dydeoxy guanosine (8-OHdG) EIA and superoxide dismutase (SOD) assay kits were obtained from Cayman Chemical Co. (Ann Arbor, MI, USA). Dihydroethidium (DHE) probes were obtained from Merck Millipore (Darmstadt, Germany). Malondialdehyde (MDA) EIA kits were obtained from BioVision, Inc, (Milpitas CA, USA). The NO ELISA kit was obtained from 4A Biotech Co., Ltd. (Beijing, China).

### Animals and treatment

Male BALB/c mice were obtained from Dossy Biological Technology Co., Ltd. (Chengdu, China). They were housed at 22±2°C in a humidity-controlled room with free access to fresh water and standard laboratory food. Following 1 week of conditioning in a 12 h light/dark cycle, eighty male mice were randomly divided into four groups: (i) Control group (saline injection); (ii) PQ + low-gAd group (PQ exposure combined with gAd pre-treatment at 500 μg/kg by tail vein injection at 12 and 36 h prior to PQ administration); (iii) PQ + high-gAd group (PQ exposure combined with gAd pre-treatment at 1000 μg/kg by tail vein injection at 12 and 36 h prior to PQ administration); and (iv) PQ (20 mg/kg) group, intraperitoneal administration (IP). The dose for high/low gAd was determined from preliminary experiments (data not shown). Mice were anesthetized with 50 mg/kg pentobarbital (Hanlim Pharm. Co. Ltd., Seoul, Korea), IP. Serum and pulmonary samples were collected at 3, 6, 12, 24 and 72 h post-PQ injection. The study was conducted in accordance with the ethical standards in the 1986 Directive 86/609/EEC, European Convention for the Protection of Vertebrate Animals Used for Experimental and other Scientific Purposes, and the Guiding Principles in the Use of Animals in Toxicology, adopted by the Society of Toxicology in 1989. The study was approved by the Committee on the Ethics of Animal Experiments of the Sichuan University (permit no. 26).

### Histopathological assessment of pulmonary tissue

Pulmonary tissue, fixed in 10% neutral-buffered formalin, was blocked in paraffin using an automated processor (Leica, Nussloch□Germany), with graded alcohol, xylene and paraffin. Sections (4 μm) were stained using hematoxylin and eosin. Digital images of the stained glass slides were obtained using a ScanScope Digital slide scanner (Aperio Technologies, Inc., Vista, CA, USA) at a magnification of ×100.

### Measurement of superoxide anion in pulmonary tissue

Lung segments obtained from control and PQ-treated mice were embedded in tissue freezing medium (Tris-buffered saline; Thermo Fisher Scientific, Waltham, MA, USA). Following freezing, 30-μm segments were cut, and mounted and cover-slipped on glass slides. Sections were treated with 2 μmol/l DHE in dimethyl sulfoxide buffer. Slides were incubated in a light-protected chamber at 37°C for 30 min. Ethidium-stained tissue was observed by inverted fluorescent microscopy at a magnification of ×100 (Olympus IX71, Olympus, Tokyo, Japan) following excitation (485 nm) and emission (535 nm). Control and PQ-treated pulmonary samples were processed and imaged in parallel.

### Measurement of pulmonary tissue MDA, SOD, NO and 8-OHdG levels, and blood 3-NT levels

Pulmonary tissue levels of MDA, SOD, NO and 8-OHdG, and blood levels of 3-NT from control and PQ-treated mice were determined using ELISA kits, according to the manufacturer’s instructions.

### Statistical analysis

All values are expressed as the mean ± standard deviation. Comparisons between groups at each time point were made by one-way analysis of variance, followed by Student-Newman-Keul’s test. P<0.05 was considered to indicate a statistically significant difference.

## Results

### gAd attenuated PQ-mediated pulmonary interstitial edema and inflammatory cell infiltration in a dose-dependent manner

**Figure 1:**
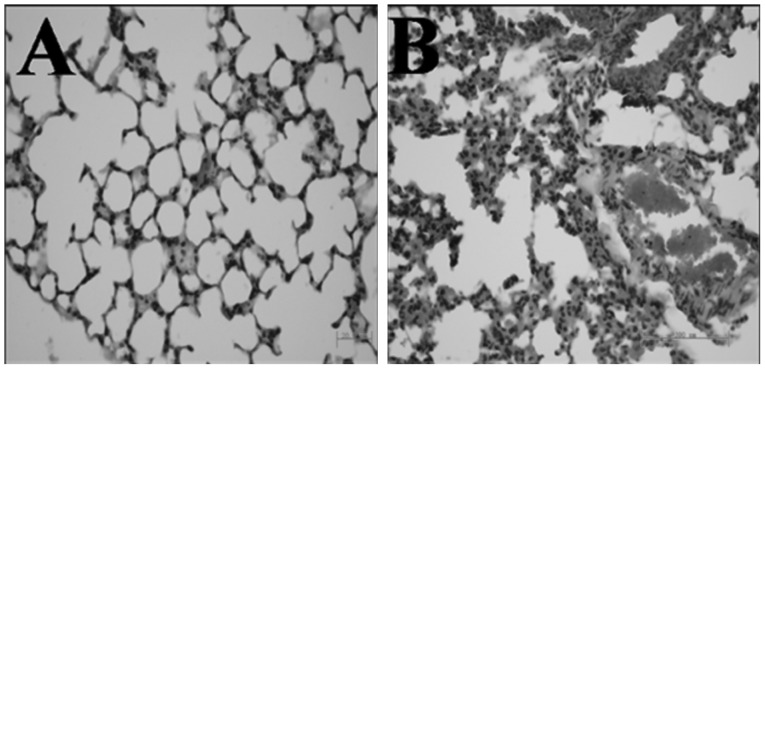
Effect of gAd pre-treatment on pulmonary edema and inflammatory cell infiltration in control animals or PQ-exposed animals, treated with vehicle or gAd (high-dose or low-dose) 24 h prior to PQ injection. (A) Control group; (B) PQ (20 mg/kg) group; (C) high-dose gAd (1000 μg/kg); and (D) low-dose gAd (500 μg/kg). Values represent the mean ± standard error of the mean of four parallel measurements. gAd, globular domain adiponectin; PQ, paraquat. Stain, hematoxylin and eosin; magnification, ×100.

Compared with the control group (Fig. 1A), PQ administration caused acute injury to pulmonary tissue, as demonstrated by the interstitial edema and inflammatory cell infiltration (lymphocytes and histiocytes) observed in the alveolar space and septum (Fig. 1B). Pre-treatment with gAd inhibited these changes (Fig. 1C and D) in a dose-dependent manner.

### gAd reduced PQ-induced pulmonary oxidative injury in a dose-dependent manner

**Figure 2:**
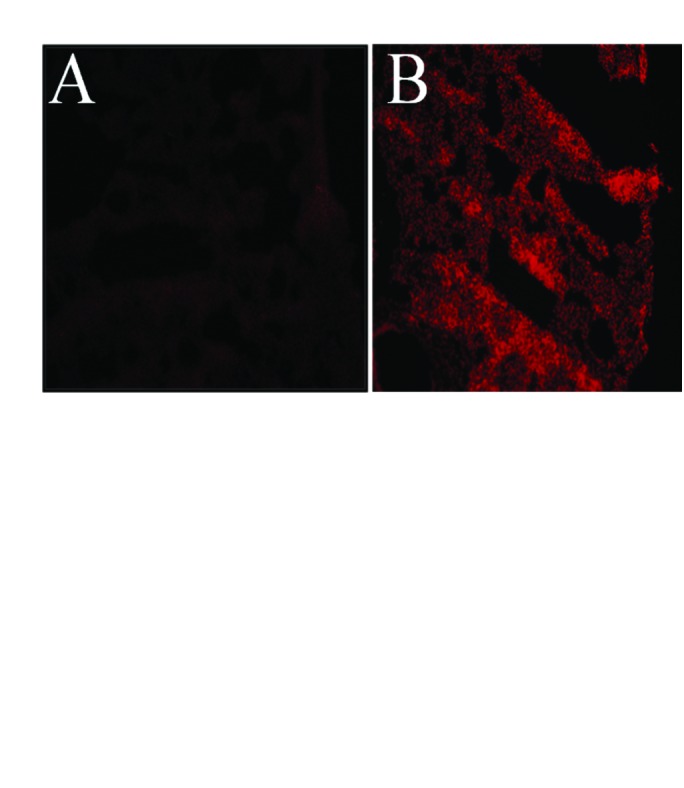
Effect of gAd pre-treatment on pulmonary superoxide anions in the lungs from control animals or PQ-exposed animals, treated with vehicle or gAd (high-dose or low-dose) 24 h prior to PQ injection. (A) Control group; (B) PQ (20 mg/kg) group; (C) high-gAd (1000 μg/kg); and (D) low-gAd (500 μg/kg). Values represent the mean ± standard error of the mean of four parallel measurements. gAd, globular domain adiponectin; PQ, paraquat. Stain, dihydroethidium; magnification, ×100.

Three series of experiments were performed to determine the effects of PQ and gAd on oxidative stress in the mouse lung. In the first series, *in situ* O_2_^•−^ generation was detected by DHE staining. Weak DHE staining was observed in the pulmonary tissue of control animals, indicating the basal pulmonic O_2_^•−^ production (Fig. 2A). The staining was significantly intensified in mice subjected to PQ administration (Fig. 2B). By contrast, gAd (at high and low doses, Fig. 2C and D) decreased this augmented staining.

**Figure 3:**
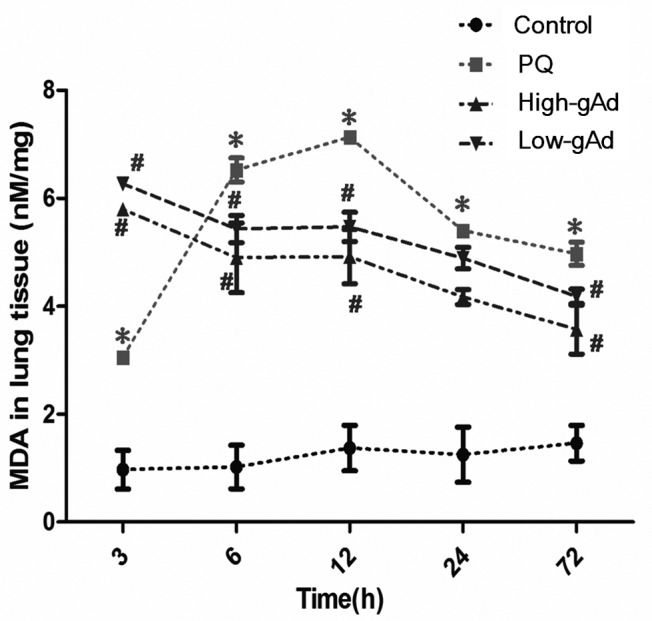
Effect of gAd pretreatment on pulmonary MDA level in control animals or PQ-exposed animals, treated with vehicle or gAd (high-dose or low-dose) at 0, 3, 6, 12, 24 and 72 h after PQ injection. MDA levels were determined by an enzyme-linked immunosorbent assay. Values represent the mean ± standard error of the mean of four parallel measurements. ^*^P<0.05, compared with controls and ^#^P<0.05, compared with the PQ group. gAd, globular domain adiponectin; MDA, malondialdehyde; PQ, paraquat.

In the second series of experiments, pulmonary tissue MDA content was found to be significantly increased in mice subjected to PQ administration at as early as 3 h following initial PQ exposure (Fig. 3). This increase was eliminated from 6 to 72 h following the initial PQ exposure, by pre-treatment with gAd.

**Figure 4:**
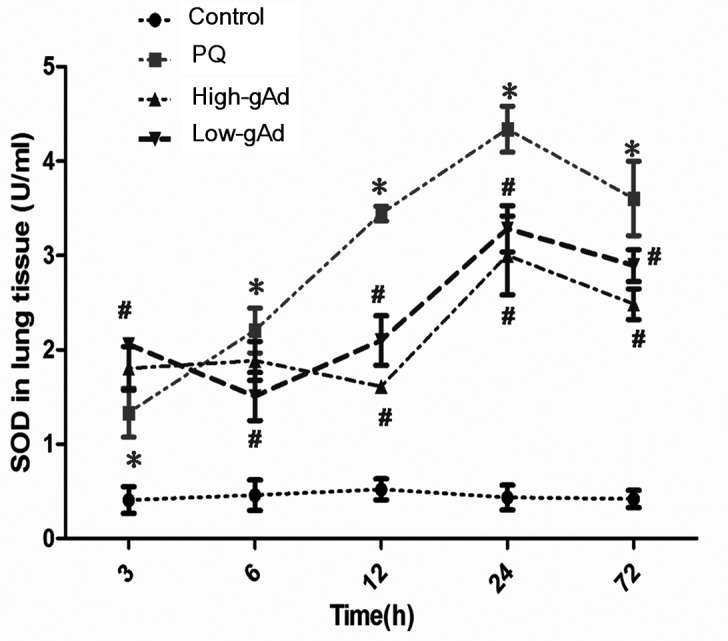
Effect of gAd pre-treatment on pulmonary SOD level in control animals or PQ-exposed animals, treated with vehicle or gAd (high-dose or low-dose) at 0, 3, 6, 12, 24 and 72 h following PQ injection. SOD levels were determined by an enzyme-linked immunosorbent assay. Values represent the mean ± standard error of the mean of four parallel measurements. ^*^P<0.05, compared with controls and ^#^P<0.05, compared with the PQ group. gAd, globular domain adiponectin; SOD, superoxide dismutase; PQ, paraquat.

In the third series of experiments, pulmonary SOD levels were found to be significantly increased at all time points measured in animals subjected to PQ administration compared with the control group (Fig. 4). Notably, the augmented SOD activity was further amplified by pre-administration of gAd at 3 h, although it was decreased from 6 to 72 h, in accordance with the trend in MDA levels.

### gAd reduced PQ-induced pulmonary nitrative injury in a dose-dependent manner

**Figure 5:**
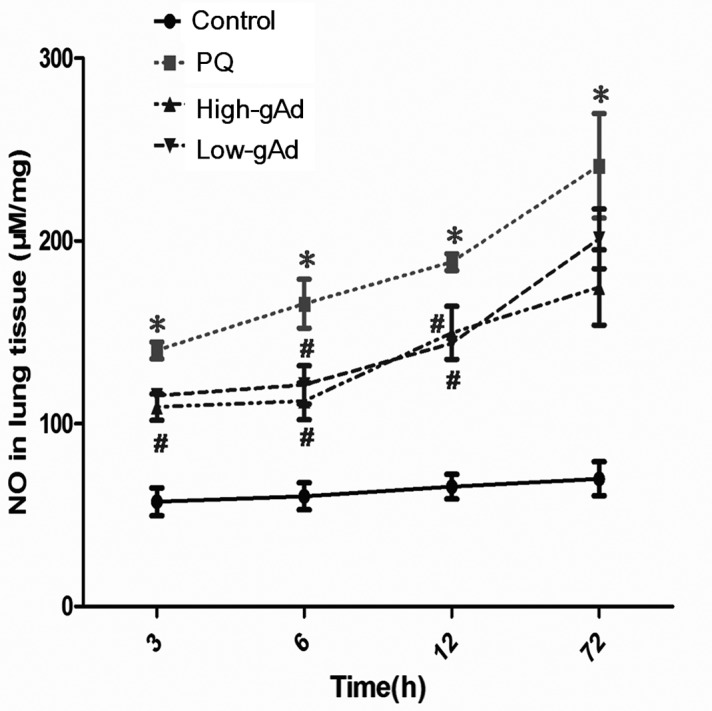
Effect of gAd pretreatment on pulmonary NO level in control animals or PQ-exposed animals, treated with vehicle or gAd (high-dose or low-dose) at 0, 3, 6, 12, 24 and 72 h following PQ injection. NO levels were determined by an enzyme-linked immunosorbent assay. Values represent the mean ± standard error of the mean of four parallel measurements. ^*^P<0.05, compared with controls and ^#^P<0.05, compared with the PQ group. gAd, globular domain adiponectin; NO, nitric oxide; PQ, paraquat.

**Figure 6:**
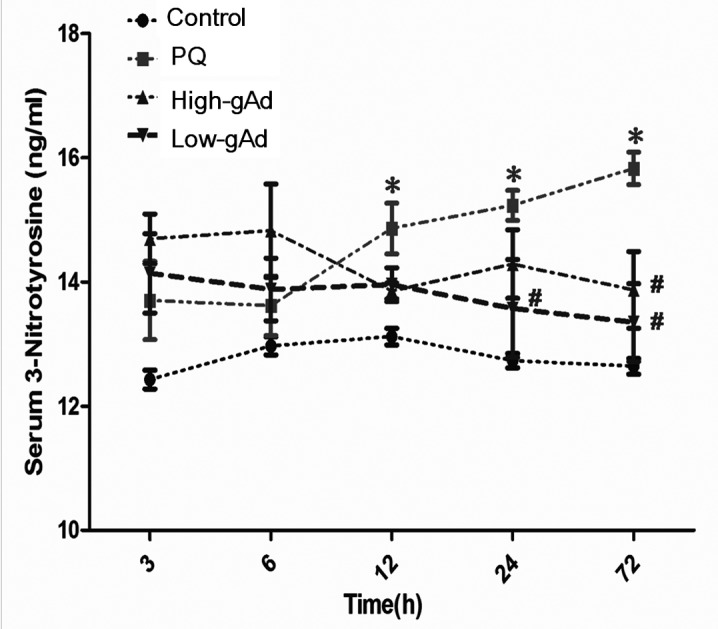
Effects of gAd pretreatment on blood 3-NT level in control animals or PQ-exposed animals, determined by an enzyme-linked immunosorbent assay at 0, 3, 6, 12, 24 and 72 h. Values represent the mean ± standard error of the mean of four parallel measurements. ^*^P<0.05, compared with controls and ^#^P<0.05, compared with the PQ group. gAd, globular domain adiponectin; 3-NT, 3-nitrotyrosine; PQ, paraquat.

NO production was significantly increased in PQ-exposed mice compared with the control group. This augmentation was significantly reduced in gAd-pre-treated mice compared with the PQ group (Fig. 5). As a consequence of the induced NO production, blood 3-NT levels were also increased in the PQ-exposed mice compared with the control group. gAd administration decreased the blood 3-NT level, particularly from 24 to 72 h following PQ-exposure (Fig. 6).

**Figure 7:**
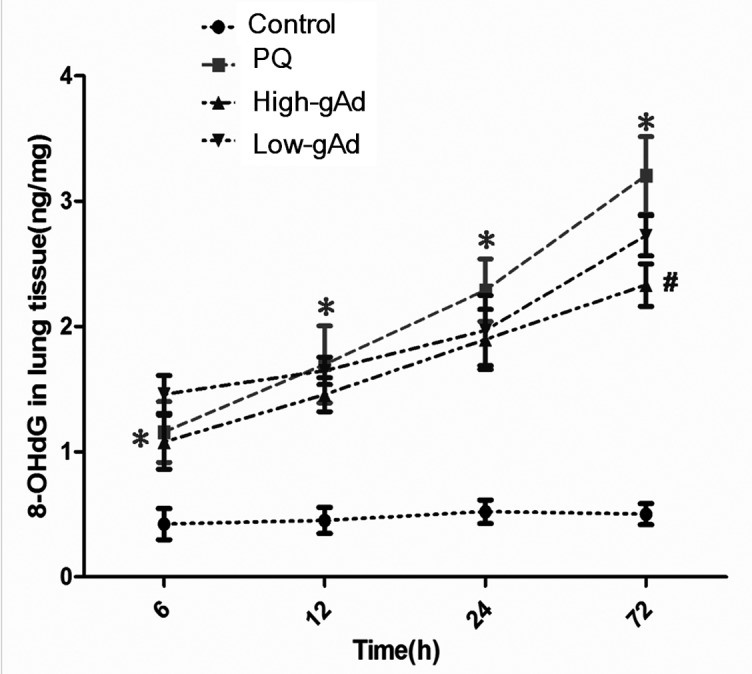
Effects of gAd pretreatment on pulmonary 8-OdGH in control animals or PQ-exposed animals, determined by an enzyme-linked immunosorbent assay at 0, 3, 6, 12, 24 and 72 h. Values represent the mean ± standard error of the mean of four parallel measurements. ^*^P<0.05, compared with controls and ^#^P<0.05, compared with the PQ group. gAd, globular domain adiponectin; 8-OdGH, 8-hydroxy-2-dydeoxy guanosine; PQ, paraquat.

Similarly, a marked increase in pulmonary 8-OHdG levels was observed in PQ-exposed mice compared with the control group. The increase was decreased by gAd pre-treatment to a degree at all time points measured, and this reduction was significant compared with the PQ group at 72 h (Fig. 7).

## Discussion

Previous experimental and clinical studies have demonstrated that PQ directly contributes to lung injury by inducing inflammation, edema and fibrosis (10–13). In the present study, gAd pre-treatment mitigated the pathological changes induced by PQ in a dose-dependent manner. This provides evidence that the adipocytokine, adiponectin, which is known to have antidiabetic (14), anti-atherogenic (15), antitumor (16) and anti-inflammatory properties (17,18), also exerts a pulmonary protective effect against PQ-induced injury.

The current study indicated that the possible mechanisms by which gAd relieves PQ-induced lung injury may be attributed to two effects. gAd protects mice against PQ-induced lung injury by attenuating oxidative stress. In addition, gAd protects against PQ-induced lung injury by mitigating nitrative stress.

PQ is a toxic herbicide that particularly affects the lungs, since the pulmonary polyamine uptake system preferentially recruits PQ, resulting in a 6–10 fold increase in lung levels compared with the plasma (19,20). PQ is known to induce alveolar collapse and fibrosis (21). Such effects are due to the imbalance between the formation and scavenging of ROS (1,2,22). ROS overproduction damages cell membranes, resulting in lipid peroxidation, for which MDA levels serve as a proxy measurement (23). SOD is a mammalian enzymatic defense system, which protects against oxidative injury by scavenging excess O_2_^−^. It also indirectly reflects the level of lipid peroxidation (24). The present study demonstrated that PQ significantly increases MDA production and activates SOD, which was consistent with a previous study (25) This suggests that PQ simultaneously stimulates oxidative stress injury and anti-oxidative defenses to ameliorate lipid peroxidation.

Consistent with previous reports, adiponectin potently scavenges free radicals, leading to the inhibition of lipid peroxidation (6). In the current study, it was demonstrated that gAd administration 12 and 36 h prior to PQ exposure significantly attenuated O_2_^−^ generation and MDA production. To the best of our knowledge, this study provides the first direct evidence supporting the hypothesis that adiponectin modulates antioxidant defense mechanisms in the lung, and could thus offer protection against PQ-induced lung damage via attenuation of oxidative and nitrative stresses.

Contrary to findings from previous studies (26,27), gAd pre-treatment at high or low doses only augmented SOD activities at 3 h following initial PQ exposure. By contrast, SOD activity was lower in the gAd groups compared with the PQ group following 6 to 72 h. There are a number of possible explanations for this effect. The most plausible of these is that gAd may stimulate anti-oxidative SOD activity in a burst-like manner, which would diminish ROS and MDA levels such that the higher levels of SOD producing the anti-oxidant effect were no longer required. An alternative explanation is that the short half-life (~13–17.5 h) of gAd in circulation (28) may limit its anti-oxidative properties over a prolonged period.

Other than ROS, reactive nitrogen species (RNS), including NO and ONOO^−^, mediate nitrative stress via the nitration of various biomolecules, including proteins, lipids and nucleic acids (29). Accumulating evidence has demonstrated that PQ augments NO and ONOO^−^ production, suggesting that RNS are also involved in PQ-mediated pulmonary injury (30–33). The current study showed that PQ significantly increased NO production, which emphasized the pathophysiological importance of NO in PQ-mediated lung injury. In contrast to its effects in an endothelial model (34), pre-treatment with gAd significantly reduced NO production in PQ-exposed mice lung. A possible explanation may be that gAd causes different effects in different cells or tissues under different stimulation.

Excess NO reacts with O_2_^−^ to produce ONOO^−^ (35,36). ONOO^−^ is a highly toxic reactive species, which nitratively modifies various proteins, for example, changing tyrosine to 3-NT (37). ONOO^−^ also injures DNA via a number of mechanisms, including the formation of 8-OHdG (38). The present study demonstrated an increase in 8-OHdG levels in lung tissue from 6 to 72 h, and increased 3-NT from 12 to 72 h, in PQ-exposed mice. These results provide additional evidence in support of the hypothesis that nitrative stress is involved in PQ-mediated injury (39). gAd pre-treatment markedly attenuated 3-NT production at 24 and 72 h, and decreased 8-OHdG after 72 h. To the best of our knowledge, this is the first evidence demonstrating that adiponectin protects against PQ-mediated lung injury by attenuating peroxynitrite-induced protein nitration and DNA damage via a reduction in nitrative stress.

In conclusion, the present study demonstrates that gAd protects against PQ-induced lung injury in a BALB/c mouse model, by attenuating oxidative/nitrative stress in a dose-dependent manner. Adiponectin is therefore a potential therapeutic agent against PQ-induced lung injury, for which no efficacious treatment currently exists. Clarification of the precise signaling mechanisms involved in the amelioration of oxidative/nitrative stress by adiponectin requires further investigation. In addition, further studies are required in order to assess the effect of gAD given with and following PQ administration, as the current study, which only examined the effect of pre-treatment with gAd, does not accurately mimic true clinical scenarios.

## Abbreviations

PQ: paraquat
gAd: globular domain adiponectin
3-NT: 3-nitrotyrosine
MDA: malondialdehyde
NO: nitric oxide
8-OHdG: 8-hydroxy-2-dydeoxy guanosine
SOD: superoxide dismutase
